# Late complications of biliopancreatic diversion in an older patient: a case report

**DOI:** 10.1186/s12877-021-02578-z

**Published:** 2021-11-04

**Authors:** Nele Steenackers, Elien Brouwers, Ann Mertens, Simon Van Cleynenbreugel, Matthias Lannoo, Johan Flamaing, Katleen Fagard

**Affiliations:** 1 Clinical and Experimental Endocrinology, Department of Chronic Diseases and Metabolism, KU Leuven, Leuven, Belgium; 2 Department of Geriatric Medicine, University Hospitals Leuven, Herestraat 49, 3000 Leuven, Belgium; 3 Department of Geriatric Medicine, Ziekenhuis Oost-Limburg, Genk, Belgium; 4 Department of Endocrinology, University Hospitals Leuven, Leuven, Belgium; 5 Department of Abdominal Surgery, University Hospitals Leuven, Leuven, Belgium

**Keywords:** Bariatric surgery, Biliopancreatic diversion, Elderly, Dumping syndrome, Neuroglycopenia, Delirium, Malnutrition, Nutritional deficiencies

## Abstract

**Background:**

In the mid-seventies, biliopancreatic diversion became popular as weight-loss surgery procedure. This bariatric procedure combines distal gastric resection and intestinal malabsorption, leading to greater weight loss and improvement of co-morbidities than other bariatric procedures. Nowadays, biliopancreatic diversion has become obsolete due to the high risk of nutritional complications. However, current patients with biliopancreatic diversions are aging. Consequently, geriatricians and general practitioners will encounter them more often and will be faced with the consequences of late complications.

**Case presentation:**

A 74-year old female presented with weakness, recurrent falls, confusion, episodes of irresponsiveness, anorexia and weight loss. Her medical history included osteoporosis, herpes encephalitis 8 years prior and a biliopancreatic diversion (Scopinaro surgery) at age 52. Cerebral imaging showed herpes sequelae without major atrophy. Delirium was diagnosed with underlying nutritional deficiencies. Biochemical screening indicated vitamin A deficiency, vitamin E deficiency, zinc deficiency and severe hypoalbuminemia. While thiamin level and fasting blood glucose were normal. However, postprandial hyperinsulinemic hypoglycemia was observed with concomitant signs of confusion and blurred consciousness. After initiating parenteral nutrition with additional micronutrient supplementation, a marked improvement was observed in cognitive and physical functioning.

**Conclusions:**

Long-term effects of biliopancreatic diversion remain relatively underreported in older patients. However, the anatomical and physiological changes of the gastrointestinal tract can contribute to the development of metabolic and nutritional complications that may culminate in cognitive impairment, functional decline and delirium. Therefore, it is warranted to evaluate the presence of metabolic disturbances and nutritional complications in older patients after biliopancreatic diversion.

## Background

**Fig. 1:**
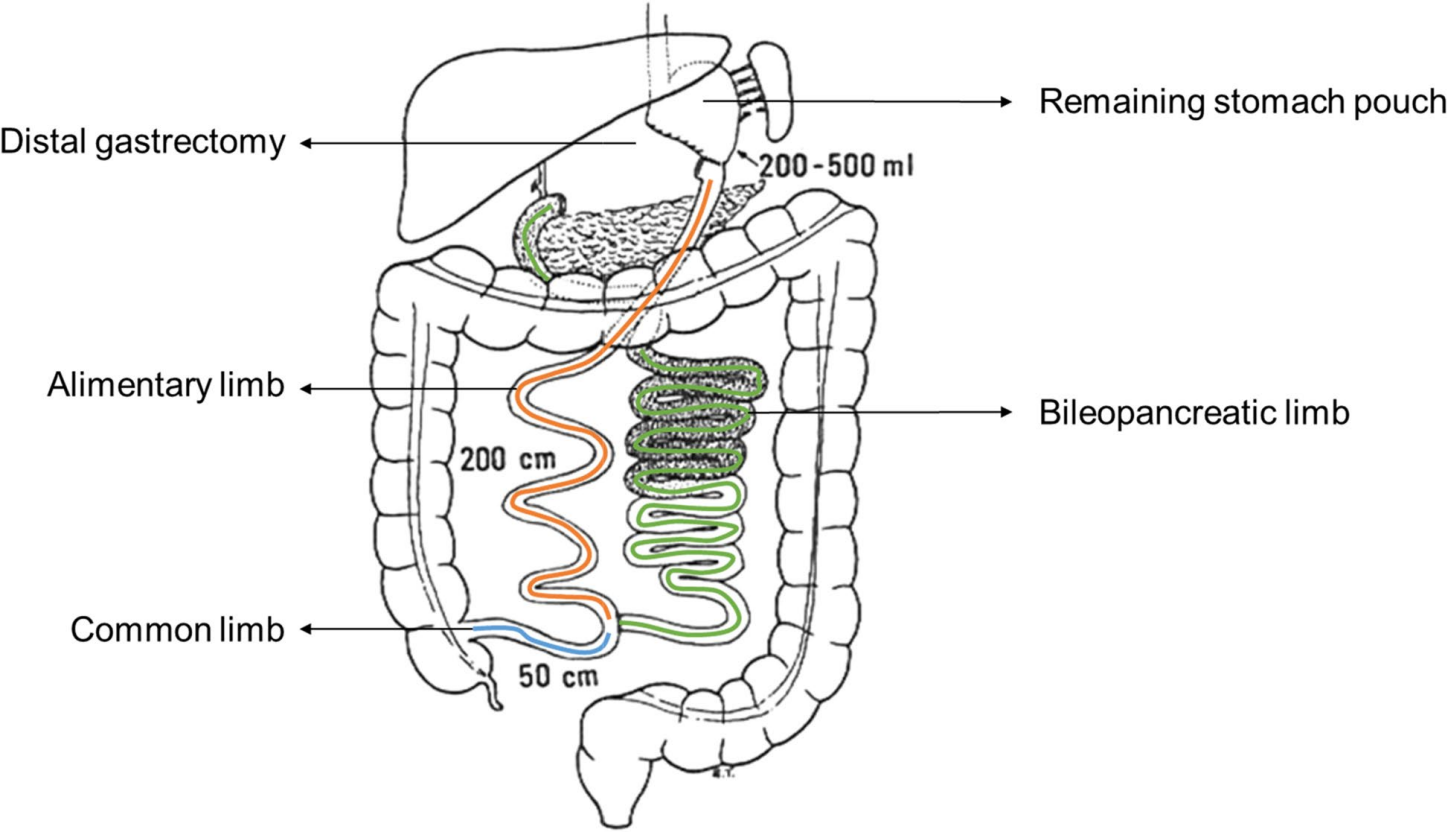
Schematic drawing from the patient’s Scopinaro surgery report

Worldwide, the prevalence of obesity has nearly tripled since 1975 [1]. This had led to an increasing demand for bariatric surgery, especially since the introduction of laparoscopy increased the safety and efficacy of bariatric procedures [2]. Different bariatric surgery procedures include Roux-en-Y gastric bypass, adjustable gastric banding, sleeve gastrectomy, biliopancreatic diversion (Scopinaro procedure) and biliopancreatic diversion with duodenal switch [3]. Among available bariatric procedures, biliopancreatic diversion was commonly performed in the previous decades [4]. This bariatric procedure combines a distal gastrectomy with a Roux-en-Y construction. The biliopancreatic limb carries pancreatic and biliary juices into the common limb, while the alimentary limb transfers the ingested food into the common limb (Fig. 1). Consequently, the Scopinaro procedure leads to intestinal malabsorption by delaying digestion until the common limb that is characterized by a length of only 50 cm [3, 4]. In general, bariatric surgery results in weight loss, improvement of almost all obesity-related co-morbidities and an increased life expectancy [5, 6]. Although biliopancreatic diversion is one of the techniques with the lowest long-term weight regain, the procedure can result in severe metabolic and nutritional complications [7, 8]. Due to the increasing demand for bariatric surgery in the last decades and increased life expectancy after surgery, late complications are now more frequently observed in older patients. Despite the important impact of late complications on older patients, they are often under- or misdiagnosed due to the presence of multiple underlying conditions. Moreover, late complications of bariatric surgery may not be recognised as one of the underlying causes of geriatric syndromes, such as falls or delirium. Here, we report the case of an older patient with a previous biliopancreatic diversion, who developed cognitive impairment, functional decline and delirium later on in life.

## Case presentation

A 74-year old woman was admitted to the geriatric department with weakness, recurrent falls, confusion, episodes of irresponsiveness, anorexia and weight loss (10 kg over 6 months). Her medical history consisted of herpes encephalitis 8 years ago, postlesional epilepsy, osteoporosis and a bariatric Scopinaro procedure at the age of 52 (Fig. 1). In the previous 3 months, she had already been hospitalised on two occasions to the geriatric department. During her first admission for recurrent falls, a multifactorial origin was assigned to the falling including (i) malabsorption with deficiencies of vitamin A and E, (ii) a urinary tract infection, and (iii) recurrent episodes of irresponsiveness that were thought to be epilepsy. However, serum levels of anti-epileptic drugs and an electroencephalogram did not confirm the epilepsy diagnosis. During this hospitalisation, there was a suspicion of early-stage dementia. Since a few months before the first hospitalisation, there were episodes of confusion, visual hallucinations and day-night reversal. However, the patient still lived alone without any professional support. She required assistance from her daughter for daily-life activities including shopping, laundry and housekeeping. During the admission, a mini-mental state examination (MMSE) revealed a MMSE-score of 28/30. While the Montreal Cognitive Assessment Examination (MOCA) revealed attention and working memory deficits with a MOCA-score of 22/30. Consequently, a transfer to the neuropsychiatric department was recommended for further diagnostic workout. Against medical advice, her daughter took her away from the hospital for a holiday abroad without continuing oral vitamin and mineral supplementation. A few weeks later, the patient was readmitted to the geriatrics department with increasing episodes of confusion and irresponsiveness, complications of being very weak and nearly without any oral intake. Laboratory blood examination revealed persisting deficiencies of vitamin A and E. While a cerebral MRI showed postherpetic sequelae in the right hippocampus and temporal lobe, limited cortico-subcortical and periventricular leuco-atrophy with multiple vasculo-ischemic supratentorial white matter lesions. Moreover, lumbar puncture findings were normal including normal levels of biomarkers for Alzheimer’s disease. Consequently, the presence of delirium was suspected. Considering the diagnosis, the patient was transferred to the neuropsychiatric unit after initiation of oral nutritional support in combination with vitamin and mineral supplementation. Here, a recurrent MMSE revealed a score of 13/30. After 2 weeks, the patient was transferred again to the geriatrics department due to persisting anorexia with difficult oral intake and a continuous decline of her cognitive and functional status. From walking with a walking aid, she had become totally dependent for her daily life activities. Moreover, she remained confused with fluctuating consciousness, which was suggestive for persistent delirium. No other concomitant symptoms were observed or reported, neither diarrhoea nor steatorrhea. However, clinical examination revealed significant hypotension (73/46 mmHg) without fever or tachycardia. In addition, the patient had a cachectic appearance and marked pitting oedema of the lower body. Her BMI amounted 20 kg/m^2^ (weight: 48.5 kg, length: 1.55 m), which is an overestimation due to the oedematous state. Her daily medication schedule consisted of vitamin A (retinyl acetate 5000 units), vitamin E (dl-alpha-tocopheryl acetate 200 units = 200 mg), iron sulphate 525 mg (105 mg elementary iron), a multivitamin B complex: B1, B2, B6 and B12 (thiamine mononitrate 250 mg, riboflavin 10 mg, pyridoxine hydrochloride 250 mg, cyanocobalamin 0.02 mg; twice daily), folic acid 0.4 mg, a calcium and vitamin D supplement (calcium carbonate 2500 mg, cholecalciferol 880 units), valproic acid (450 mg; twice daily) and lamotrigine (100 mg; twice daily). Despite the oral nutritional support and supplementation, recurrent laboratory blood examination revealed persistent nutritional deficits including severe hypalbuminaemia (16 g/l; normal range: 35–52), vitamin A deficiency (115 μg/l; normal range: 300–650), vitamin E deficiency (1.5 mg/l; normal range: 5–20) and zinc deficiency (36 μg/dl; normal range: 80–120). While other nutritional markers were within normal range including calcium, 25-OH vitamin D, prothrombin time, folic acid and iron panel (intravenous iron and vitamin K had been administered during the first admission). Moreover, vitamin B1 and B12 levels were high. Unfortunately, copper was not measured on admission. There was no proteinuria and the creatinine level was within the low normal range. Again, plasma antiepileptic drug concentrations were normal. Venous ammonia was normal. Regarding glycaemic control, a glycated haemoglobin of 4.0% (4.0–6.0%), a morning glucose of 94 mg/dl and at the same time, a slightly elevated C-peptide was observed. However, recurrent hypoglycaemia up to 43 mg/dl was observed after her meals and was accompanied by confusion and blurred consciousness. After a multidisciplinary consultation between the geriatrics, endocrinology and abdominal surgery department, treatment with parenteral nutrition was initiated in combination with oral micronutrient supplementation. In the course of the next 3 weeks, there was a clear improvement in her cognitive status and daily functioning. After 4 weeks, her MMSE score increased again up to 28/30. In addition, weight gain was observed (5 kg) with an increase in albumin level up to 30.8 g/l. Consequently, she was transferred to a rehabilitation centre while continuing parenteral nutrition for four more weeks. On day seven of the rehabilitation, her MOCA score was 25/30. During the 4 weeks, parenteral nutrition was gradually combined with an oral diet, consisting of small frequent meals rich in proteins, complex carbohydrates, fibres and a limited amount of fat. During this period, her daily functioning improved significantly to a point where she regained her independence in daily life activities except for the use of a walking aid. After her stay in the rehabilitation centre, revision surgery was planned to reduce malabsorption by lengthening the common limb. Unfortunately, she developed catheter sepsis in the rehabilitation centre during the fourth week. Consequently, she was transferred to a high dependency unit were she died from subsequent *Clostridium difficile* pseudomembranous colitis.

## Discussion and conclusions

Since the obesity epidemic and introduction of laparoscopic surgery, bariatric surgery has become more and more popular [2]. As a result, geriatricians will be increasingly confronted with the late complications of bariatric surgery. However, there is a risk that these late complications will not be recognized and addressed in time due to the overlapping presence of age-related decline and comorbid medical issues in older patients. In this case, we report the development of cognitive impairment, functional decline and delirium in an older patient that underwent a biliopancreatic diversion more than 20 years ago. At first, underlying dementia was suspected. However, later on she was diagnosed with multifactorial delirium. Sequelae of herpes encephalitis in combination with nutritional complications were considered as the main predisposing factors. Later in the disease process, the potential contribution of post-prandial hypoglycaemia was recognized. To alleviate these problems, oral nutritional support was initiated under the form of nutritional drinks and supplementation. However, a favourable evolution of her cognitive status and daily functioning was only observed after switching to parenteral nutrition in combination with nutritional supplementation.

Despite a dearth of studies examining late complications in older patients after bariatric surgery, a multifactorial origin can be described to the development of the observed neuropsychological complications in combination with her medical history [9]. The Scopinaro procedure combines a distal gastrectomy with a Roux-en-y reconstruction of the small intestine and a very short common limb [4]. These anatomical alterations can lead to challenging consequences including (i) metabolic disturbances, (ii) severe malnutrition and (iii) nutritional deficiencies [10]. When not addressed in time, neuropsychological complications can develop [10, 11].

Regarding metabolic disturbances, the episodes of hypoglycaemia could have contributed to the patient’s confusional episodes and fluctuations in consciousness. Postprandial hyperinsulinemic hypoglycaemia is a late metabolic complication of bariatric surgery, which has historically been referred to as late dumping syndrome [12, 13]. Postprandial hypoglycaemia is caused by a rapid emptying of the gastric remnant, followed by an accelerated entry and absorption of nutrients in the small intestine. The former results in a rapid and distinct secretion of incretins and thus insulin, that is not balanced by a continuous glucose delivery and leads to hypoglycaemia [14]. Symptoms of postprandial hyperinsulinemic hypoglycaemia can develop months to years after surgery, but occur within one to 3 h after meal intake [15]. Hypoglycaemia symptoms are categorized as autonomic, including tremor, sweating and palpitation, or neuroglycopenic, including confusion, weakness, light-headedness, dizziness, blurred vision, disorientation, and eventually loss of consciousness [13, 16, 17]. Consequently, screening is of utmost importance and can be performed by means of the following criteria: (a) the presence of neuroglycopenic symptoms beyond 1 year after surgery, (b) normal fasting glucose and insulin levels, (c) correlation of symptoms with hypoglycaemia, followed by a spontaneous resolution of hypoglycaemia, and (d) a positive provocative test (e.g. mixed meal tolerance test) [13]. In this case, the presence of postprandial hyperinsulinemic hypoglycaemia was initially undiagnosed due to the similarity between the neuroglycopenic symptoms and the mental symptoms of delirium.

Regarding malnutrition, the combination of hypoalbuminemia, oedema and weight loss should raise suspicion for the presence of severe protein calorie malnutrition after biliopancreatic diversion [18]. Symptoms of protein calorie malnutrition include general weakness, fatigue and hair loss and, in more severe cases, may lead to oedema, organ failure and death [19, 20]. Severe protein calorie malnutrition is more frequently observed in patients with a short common limb, which was the case for this patient. Next to protein calorie malnutrition, nutritional deficiencies are common with a reported prevalence up to 90% after biliopancreatic diversion [21]. Micronutrient deficiencies and associated symptoms after biliopancreatic diversion are summarized in Table 1 [10, 22–25]. Different factors contribute to the development of nutritional deficiencies after bariatric surgery including a reduced gastric acid secretion, a more rapid intestinal transit and a delayed inlet of pancreatic enzymes and biliary secretions. In addition, a large area for absorption with region-specific transporters is bypassed resulting in a short absorption surface of the common limb. Together, these gastrointestinal physiological alterations can contribute to micronutrient malabsorption [26]. Moreover, an excessive reduction i caloric intake, food intolerance, non-compliance to nutritional supplementation or short-intestinal bacterial overgrowth can contribute or even aggravate the nutritional status after surgery [22]. The presence and type of nutritional deficiencies depends largely on the type of bariatric procedure with deficiencies in fat-soluble vitamins (e.g. vitamin A, D, E, and K), calcium, copper and zinc being commonly reported after a malabsorptive biliopancreatic diversion [21, 27]. When considering the neuropsychological symptoms of our case, a manifestation of different nutritional deficiencies could have contributed including deficiencies of water-soluble vitamins (e.g. B1 and B12), fat-soluble vitamins (e.g. vitamin A and E) and minerals (e.g. zinc or copper). However, laboratory blood examination revealed normal levels of vitamin B1 and B12. The former excluded both vitamin B1 deficiency with Wernicke encephalopathy and vitamin B12 deficiency with neurological manifestations as potential contributing factors [28, 29]. Nonetheless, the patient suffered from vitamin A and E deficiency throughout the hospitalization. While vitamin A deficiency is generally associated with ocular complications after bariatric surgery, it may have contributed to the cognitive decline due to its role in regulating neuroplasticity [30–32]. Regarding the presence of vitamin E deficiency, clinical symptoms are generally rare but are associated with neurological manifestations [30, 33]. Moreover, zinc deficiency was observed and could have contributed to the neuropsychological symptoms due to its neuroprotective activity when present in normal physiological concentration [23, 34]. Unfortunately, the contribution of copper deficiency cannot be excluded due to the absence of a recent blood examination. However, a previous case report described the presence of neurological symptoms in patients with hypocupraemia after gastric bypass [35]. The therapeutic approach to resolve malnutrition and nutritional deficiencies depends on its severity and the degree of malabsorption. In mild cases, oral nutritional supplementation might be sufficient [36]. In more severe cases, enteral or parenteral nutritional support should be considered with additional intravenous or intramuscular micronutrient supplementation. In case of enteral or parenteral nutrition, sufficient attention is needed to avoid refeeding syndrome, malabsorptive diarrhoea and catheter-related blood stream infections [20, 22]. Refeeding syndrome may cause additional complications including severe thiamine deficiency, fluid and electrolyte disorders (e.g. hypophosphatemia, hypokalaemia and hypomagnesaemia), which should be monitored closely and supplemented [37]. In life-threatening cases with persistent malnutrition, surgical lengthening of the common limb or surgical revision should be considered.

**Table 1:** Common micronutrient deficiencies, their symptoms, and guidance for supplementation after biliopancreatic diversion

Deficiency	Signs and symptoms [10, 22–24]	Supplementation in case of deficiency^a^ [25]
Fat soluble vitamins^b^		
Vitamin A	Night blindness	No corneal changes: 10,000–25,000 IU per day orally^c^; corneal changes: 50,000–100,000 IU administered intramuscularly (3 days) followed by 50,000 IU intramuscularly (two weeks).
Vitamin D	Osteoporosis, fractures	3000–6000 IU per day orally
Vitamin E	Ataxia, loss of vibration or position sense, muscle weakness	Optimal therapeutic dose undefined
Vitamin K	Coagulation disorder (bleeding or bruising)	1–2 mg per day orally in case of acute malabsorption or 1–2 mg per week intravenously in case of chronic malabsorption
Water soluble vitamins		
Vitamin B1 (thiamine)	Gastro-enterologic: nausea, vomiting; Wet beriberi: cardiovascular symptoms; Dry beriberi: neurological symptoms (Wernicke-Korsakov syndrome**)**	Orally: 100 mg (2–3x per day); Intravenously: 200 mg (3x per day) or 500 mg (1-2x per day) until symptoms resolve and consider oral therapy afterwards (100 mg); Intramuscularly: 250 mg (1x per day during 3–5 days) or 100–250 mg (1x per month).
Vitamin B9 (folic acid)	Fatigue, anaemia, cognitive impairment, depression	1000 μg per day orally^d^
Vitamin B12 (cobalamin)	Neuropathy, muscle weakness, fatigue, anaemia, mood disorders	1000 μg orally or intramuscularly
Trace metals		
Iron	Fatigue, microcytic anaemia, hair loss, brittle nails, angular cheilosis	150–200 mg of elemental iron orally^e,^ in non-responders intravenous iron infusion should be considered
Zinc	Diarrhoea, **anaemia,** hair loss, glossitis, hypogeusia, delayed wound healing, skin lesions **and mental abnormalities**	Optimal therapeutic dose undefined
Copper	Painful neuropathy, anaemia, neutropenia, optic neuropathy, fatigue, iron deficiency	Mild to moderate deficiency: 3–8 mg per day orally; Severe: 2–4 mg per day intravenously

^a^ daily required dose in case of deficiency detected by biochemical monitoring; ^b^ titrate individually, higher doses of substitution may be required following malabsorptive procedures; ^c^ Caution to avoid toxicity, especially in patients with kidney disease who have reduced vitamin A clearance; ^d^ more than 1 mg not recommended because of the potential masking of vitamin B12 deficiency; ^e^ separate from calcium supplements and gastric acid-reducing medications, association of vitamin C enhances ferric iron absorption

To the best of our knowledge, this is the first case report that describes the medical course of a geriatric patient that developed neuropsychological symptoms due to late complications of biliopancreatic diversion. It is plausible to assume that the neuropsychological complications were caused by multiple contributing factors including the presence of metabolic disturbances, severe protein calorie malnutrition, various nutritional deficiencies and her medical history. Consequently, the presence of metabolic and nutritional complications should always be screened when an older patient develops neuropsychological symptoms following biliopancreatic diversion, even if they are considered rare. To monitor the metabolic and nutritional status after bariatric surgery, different societies have provided guidelines including (i) periodic laboratory screening to detect the presence of nutritional deficiencies, (ii) prophylactic nutritional supplementation and (iii) additional therapeutic supplementation according to the diagnosed nutritional deficiencies [38–40]. Although nutritional screening and supplementation is advised, the recommendations vary regarding the timing of screening and the type and amount of nutritional supplementation. Moreover, the underlying evidence for these guidelines is rather underdeveloped [41]. In the meantime, these local guidelines should be applied. A summary of the screening and supplementation guidelines in provided in Table 1. Importantly, sufficient attention should be devoted to the medical course of each patient individually. In older patients, one should be aware that there is a lower compliance to recommendations and a higher drop out from follow-up. Consequently, there is a need for more awareness as symptoms can be misinterpreted due to the overlapping presence of age-related decline and comorbid medical issues. Moreover, the effects of chronic malabsorption may be exacerbated by the presence of frailty or malnutrition.

In summary, due to the increasing popularity of bariatric surgery and the ageing of patients, geriatricians and general practitioners will encounter more older patients suffering with the consequences of late complications. Nowadays, long-term effects of biliopancreatic diversion remain relatively underreported in older patients. However, the anatomical and physiological changes of the gastrointestinal tract can contribute to the development of metabolic and nutritional complications that may culminate in cognitive impairment, functional decline and delirium. Therefore, it is warranted to actively evaluate the presence of metabolic disturbances, malnutrition and nutritional deficiencies in older patients after biliopancreatic diversion.

## Data Availability

Data sharing is not applicable as no datasets were generated. All relevant data has been presented in the manuscript. Further inquiry can be directed to the corresponding author.

## Abbreviations

BMI: Body mass index
MMSE: Mini-mental state examination
MOCA: Montreal cognitive assessment examination
